# Pressure-induced hydrogen-dominant high-temperature superconductors

**DOI:** 10.1093/nsr/nwae004

**Published:** 2024-01-04

**Authors:** Ho-kwang Mao

**Affiliations:** Shanghai Advanced Research in Physical Sciences, Shanghai, China; Center for High Pressure Science and Technology Advanced Research, Beijing, China

## Abstract

The century-old pursuit of room temperature superconductivity has finally been reached in highly compressed hydrogen-dominant compounds. Future efforts will be focused on understanding the high-pressure hydrogen physics and ambient-pressure applications.

Recent studies of hydrogen-dominant (superhydride) materials have led to putative discoveries of near-room temperature superconductivity at high pressures. The idea started more than half-a-century ago as a by-product of the quest for metallic hydrogen for which a natural consequence of its very high Debye temperature and phonon frequency due to the very low mass of the hydrogen atoms would lead to a high superconducting critical temperature (*T_c_*) in weak coupling Bardeen-Cooper-Schrieffer (BCS) expression. Early pursuits were unsuccessful in that pressure-induced hydrogen-dominant compounds were either non-superconducting or superconducting with a low *T_c_*, until the major theoretical and experimental breakthroughs of the past decade. Here I will summarize the key discoveries, pitfalls, the necessary technological advancement needed for future explorations, and prospects of practical applications.

The turning point came in 2012 [1] when first-principles calculations by Yanming Ma's group at Jilin University using the CALYPSO code developed by the same group predicted that CaH_6_ crystalizes into a sodalite structure above 150 GPa. In the new structure, hydrogen atoms forming ‘clathrate’ cages around large calcium atoms have the unique advantage of reaching an unprecedented high *T_c_* of 220–235 K. Ma's group further expanded the calculations to cover all rare-earth

element superhydrides with variable hydrogen stoichiometries [2,3], and found the general trend of clathrate hydrides with unusually high *T_c_* at high pressures (Fig. 1). Based on the theoretical prediction, Mikhail Eremets’ group at MPI, Mainz, Germany, experimentally synthesized a face-centered-cubic clathrate structured LaH_10_ that showed a record-breaking superconductivity *T_c_* of 250 K at 170 GPa [4], and the discovery was immediately confirmed by an independent study [5].

Subsequently, the field shows an explosive growth with experimental discoveries of numerous high-pressure high-*T_c_* superhydrides all over the Periodic Table that shared the common rule of hydrogen forming a clathrate cage around metal cations. Finally, the prediction by Ma's group was confirmed by their successful synthesis of CaH_6_ with the originally predicted [1] sodalite clathrate structure and *T_c_* of 215 K at 172 GPa [6]. At the same time, Changqing Jin's group at the Institute of Physics, Chinese Academy of Sciences, also synthesized CaH_6_ and demonstrated its high *T_c_* at high pressures [7]. These are astonishing agreements between theory and experiments.

**Figure 1.**
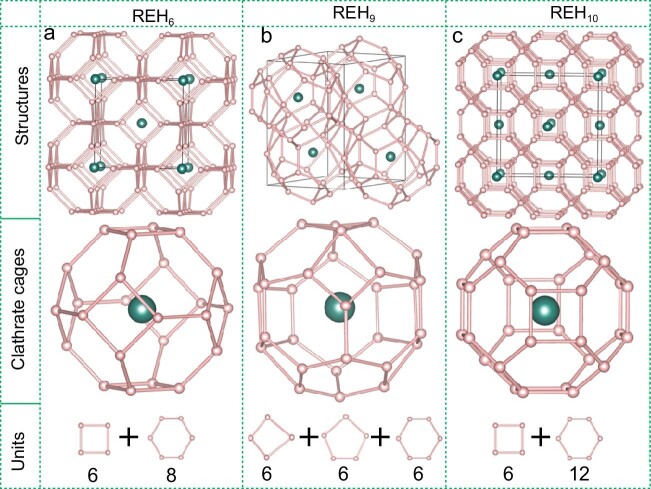
Clathrate structures of (a) REH_6_, (b) REH_9_, and (c) REH_10_. Small spheres indicate hydrogen atoms forming cages around the large rare earth (RE) atoms. From Fig. 2 of Ref. [2].

The great scientific success and tremendous public attention in room-temperature superconductors as a dream material have also inspired in-depth analyses and scrutiny in all reported experimental details, and resulted in retraction of the room-*T_c_* report [8] from Ranga Dias’ group due to its questionable background subtractions applied to the raw data used to generate the magnetic susceptibility plots at high pressure. Another publication of ambient *T_c_* superconductivity in a nitrogen-doped lutetium hydride at only 1 GPa pressure reported by the same group [9] has stimulated even greater general interest and swift testing that refuted its sample identification and evidence of superconductivity [10]. Overall, these lessons have enhanced the public awareness and understanding of the superconductivity and high-pressure sciences, as well as reconfirming the importance of robust sample characterizations, criteria of superconductivity, and reproducibility tests by independent groups.

So far, the near-room temperature *T_c_* has been robustly demonstrated in high-pressure hydrogen-dominant clathrate structures predicted by theories [2]. The key issue is that the hydrogen must be in the clathrate structure (Fig. 1); in fact, a very high ratio of hydrogen not in the cage structure, such as XeH_14_, does not form high *T_c_* superconductors even when compressed to 300 GPa. However, the hydrogen positions in the clathrates have only been predicted by theory, but have not yet been observed experimentally. Further scientific understandings of the superhydride physics require direct experimental characterizations of their atomic, electronic, and phonon structures beyond the electrical and magnetic measurements of superconductivity, and are waiting for unprecedented advancement of critically missing diagnostic techniques capable of testing the predicted hydrogen structure. A new generation of synchrotron X-ray techniques, including X-ray diffraction that provides the crucial crystal structure information, the eV-resolution inelastic X-ray spectroscopy that defines the electronic structure and monitors the bandgap closure, and the meV-resolution inelastic X-ray spectroscopy that determines phonon dynamics has hence enabled quantitative high-pressure studies of hydrogen which has the weakest known X-ray scatter and was previously considered ‘invisible’ by X-ray. Successful integration of high-pressure diamond-anvil cells with nuclear magnetic resonance spectroscopy [11] has brought one of the most powerful techniques for monitoring the behavior of hydrogen nuclear spin and structural variation at high pressures. Now the time is ripe for comprehensive investigation of hydrogen which holds the key for rich superconducting physics at high pressures.

A room-temperature superconductor is a dream material only if it could be used at ambient pressure conditions. Such a prerequisite is contradictory with our fundamental understanding of hydrogen-dominant high-*T_c_* superhydrides that require enormous pressures for dissociating the H_2_ diatomic bonds to form the calculated hydrogen atomic cages [2]. Current research community efforts are thus devoted to casting a wide combinatorial net of two-, three-, or four-component superhydrides, using chemical pressures [12] to maximize the *T_c_* while minimizing the required external pressure. Another perspective is to seal the pressure and high-*T_c_* superhydrides in superhard capsules [13] to preserve the pressure and the corresponding superior properties for ambient applications. The final goal of room-*T_c_* superconductors may be met halfway by combined progresses from both directions.
